# *Carya luodianensis* (Juglandaceae), a New Species From Guizhou Province, Southern China, Revealed by Morphological and Plastid Evidence

**DOI:** 10.1002/ece3.73047

**Published:** 2026-03-10

**Authors:** Yan‐Bing Yang, Chong‐Yi Yang, Wei‐Hao Yao, Bing Yang, Lang Huang, Ming‐Tai An, Guo‐Bing Jiang, Hong Luo, He Li

**Affiliations:** ^1^ Guizhou Academy of Forestry Guiyang Guizhou China; ^2^ Fanjingshan National Observation and Research Station of Chinese Forest Ecosystem Jiangkou Guizhou China; ^3^ College of Life Science Guizhou University Guiyang Guizhou China; ^4^ College of Forestry Guizhou University Guiyang Guizhou China; ^5^ Center for Biodiversity and Natural Conservation Guizhou University Guiyang Guizhou China

**Keywords:** *Carya*, Guizhou, new taxon, phylogeny, plastome

## Abstract

*Carya luodianensis* Y.B. Yang & M.T. An (Juglandaceae), a new species from Luodian County, Guizhou Province, China, is described and illustrated based on morphological and molecular data. Morphologically, this species resembles *C*. *kweichowensis* but can be easily distinguished by its rusty brown buds, 5–7 leaflets, a greater number of lateral veins (12–21 pairs), pubescent anthers, and nut shell bearing four faint longitudinal ridges. Molecular phylogenetic analysis based on complete chloroplast genome sequences indicates that *C*. *luodianensis* is sister to *C*. *kweichowensis*. The plastome of *C*. *luodianensis*, with a total length of 175,255 bp, exhibits a typical quadripartite structure and consists of 78 protein‐coding genes (PCGs), 30 transfer RNA (tRNA) genes, and four ribosomal RNA (rRNA) genes. Notably, a marked expansion of the inverted repeat (IR) region (40,956 bp) and contraction of the small single‐copy (SSC) region (3553 bp) are detected, accompanied by the translocation of several genes from the SSC region to the IR regions, resulting in increased gene copy numbers and reduced GC content. Comparative plastome analyses further reveal the conservation and differentiation between *C. luodianensis* and its closely related species, particularly in genome size, gene order, and repeat sequence distribution. Collectively, the unique morphological traits, plastid genome features, and phylogenetic placement strongly support the recognition of *C. luodianensis* as a distinct new species and highlight its evolutionary divergence within *Carya*.

## Graphical abstract

*Carya luodianensis* (Juglandaceae), a new species, is described and illustrated. We combined morphological characteristics, comparative plastome analyses, and phylogenetic analysis, all of which support the recognition of *C. luodianensis* as a new species and highlight its evolutionary divergence within Carya.

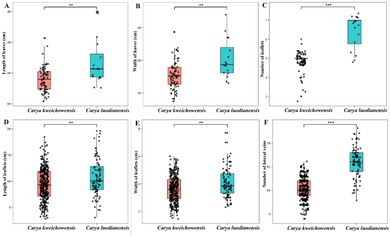

## 1 Introduction

*Carya* Nutt., the second‐largest genus within the Juglandaceae, comprises approximately 20 recognized species that exhibit a classic disjunct distribution between eastern North America and East Asia (Stone and Whittemore 1997; Lu et al. 1999; Zhang et al. 2013; Xi et al. 2022). Many species of this genus are of significant economic importance due to their production of high‐quality timber and/or nutrient‐rich nuts (Chang and Lu 1979; Huang et al. 2019). Taxonomically, *Carya* is divided into three sections based primarily on differences in the presence, number, and arrangement of bud scales, namely sect. *Apocarya* C.DC., sect. *Carya*, and sect. *Sinocarya* Cheng & R.H. Chang (Manning 1978; Chang and Lu 1979; Grauke 2003). This sectional delimitation corresponds closely with biogeographic patterns, with sect. *Carya* and *Apocarya* endemic to North America and sect. *Sinocarya* restricted to East Asia.

In China, four native species and one introduced species of *Carya* have traditionally been recognized: *C. cathayensis* Sarg., *C. hunanensis* W.C. Cheng & R.H. Chang ex R.H. Zhang & A.M. Lu, *C. kweichowensis* Kuang & A.M. Lu ex Chang & Lu, *C. tonkinensis* Lecomte, and the introduced *C. illinoinensis* (Wangenheim) K. Koch (Lu et al. 1999). Recent taxonomic revisions based on molecular and morphological evidence have expanded this circumscription. *Annamocarya sinensis* (Dode) J.‐F. Leroy was transferred to *Carya* and *C. dabeishansis* Y.Z. Hsu & N.C. Tao was elevated to species rank, rather than treated as a variant of *C. cathayensis* (Manos et al. 2007; Zhang et al. 2013; Huang et al. 2019; Luo et al. 2022; Xi et al. 2022). Moreover, *C. poilanei* was rediscovered in Yunnan Province after a 63‐year absence (Zhang et al. 2022), and a new species, *C. luana* C.Y. Deng & X.G. Xiang, was recently described from Guizhou Province, China (Xiang et al. 2023). Consequently, eight native species of *Carya* are currently recognized in China. With the exception of *C. poilanei* , *C. sinensis* , and *C. tonkinensis* , all are endemic to China, and most species are rare, exhibiting highly restricted and non‐overlapping distribution ranges (Manning 1963; Chang and Lu 1979; Lu et al. 1999; Grauke and Mendoza‐Herrera 2012; Srisanga 2017; Xi et al. 2022; Zhang et al. 2022; Xiang et al. 2023).

In September 2023, during field investigations in the limestone mountainous region of Luodian County, Guizhou Province, China, we collected a distinctive specimen of *Carya*. Based on its limestone habitat, elliptic to elliptic‐lanceolate leaflet blades, and smooth husks, the specimen was initially identified as *C. kweichowensis*. However, detailed morphological examination combined with phylogenetic analyses using chloroplast genome data revealed substantial differentiation between this taxon and *C. kweichowensis*, as well as from all other known congeners. Moreover, the chloroplast genome exhibited distinct structural features compared with other *Carya* species. Taken together, these findings support its recognition as a new species. In this study, we formally describe it as *C. luodianensis*.

## 2 Materials and Methods

### 2.1 Morphological Studies

**FIGURE 1:**
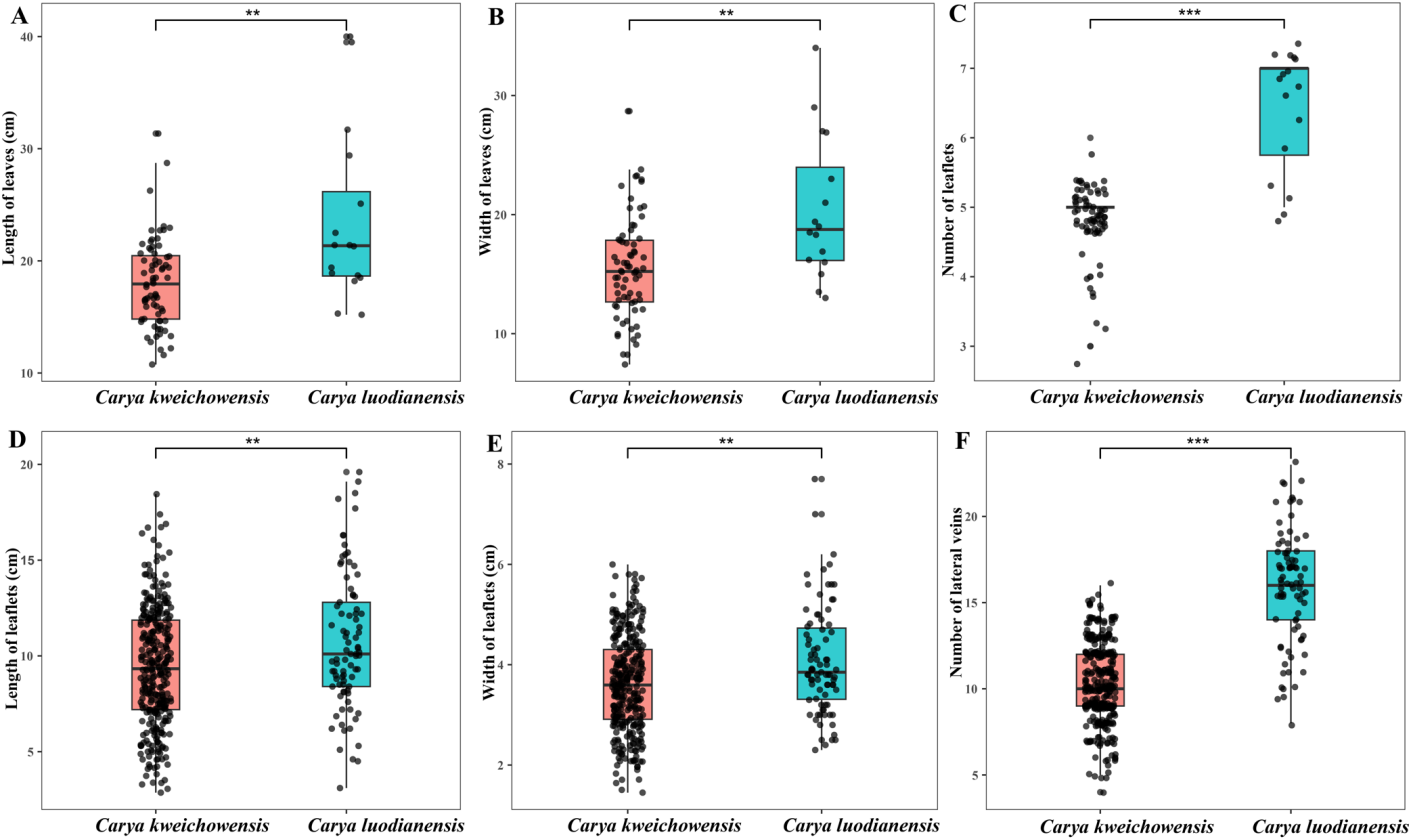
The variation in six traits between *Carya luodianensis* and *Carya kweichowensis*. (A) Length of leaves. (B) Width of leaves. (C) Number of leaflets. (D) Length of leaflets. (E) Width of leaflets. (F) Number of lateral veins. “**” 1% level of significance and “***” 0.1% level of significance.

Morphological characteristics of the new species and its similar species *Carya kweichowensis* were observed and measured using a ruler based on herbarium dried specimens and/or live plants in the field. Morphological terms follow Xiang et al. (2023). Comparisons with morphologically similar species were carried out using the specimens deposited in Herbarium of the Institute of Botany, Chinese Academy of Sciences (PE), Herbarium of Kunming Institute of Botany, Chinese Academy of Sciences (KUN), Herbarium, Guizhou Institute of Biology (HGAS), Dendrological Herbarium, College of Forestry, Guizhou University (GZAC), and Herbarium, Guizhou Academy of Forestry (GF), as well as digitized specimens from Chinese Virtual Herbarium (https://www.cvh.ac.cn/) and JSTOR Global Plants (https://plants.jstor.org/). Totally, 43 specimens of *C. kweichowensis*, *C. luana*, and *C. tonkinensis* were examined. Seventeen diagnostic characteristics involved in bud, leaflet, vein, inflorescence anther, and nut shell were selected to conduct the comparisons (Table 1). In addition, we conducted independent samples *t*‐test of six traits (leaves length, leaves width, number of leaflets, lesflets length, leaflets width, and number of lateral veins) of 34 specimens of new species and *C. kweichowensis* (Figure 1).

**TABLE 1:** Comparison of morphological traits of *Carya luodianensis* and its relatives.

Character	*Carya luodianensis*	*Carya kweichowensis*	*Carya luana*	*Carya tonkinensis*
Habit	Semi‐evergreen	Semi‐evergreen	Semi‐evergreen	Deciduous
Bud	Rusty brown	Brownish black	Rusty brown	Rusty brown
Rachis	Puberulent	Glabrous	Glabrous	Puberulent
Leaves	18–40 cm, without scales	11–20 cm, without scales	8–30 cm, without scales	15–25 cm, with abundant, peltate scales
Number of leaflets	5–7	5	3–7	5–7
Leaflets blade	Elliptic to elliptic‐lanceolate; terminal leaflets 15–20 × 4.5–6.5 cm; lateral leaflets 8–18 × 3.5–5 cm	Elliptic to elliptic‐lanceolate, (3‐)6–14 × 2–7 cm	Lanceolate or ovate‐lanceolate; terminal leaflets 10.0–16.0 × 2.2–3.3 cm; lateral leaflets 5.0–8.5 × 1.5–2.1 cm	Ovate‐lanceolate to elliptic‐lanceolate or obovate‐lanceolate, 7.0–18.0 × 2.0–6.0 cm
Lateral veins	12–21	11–13	11–18	20–25
Male spikes	3–4	1–3	1–3	2–3
Peduncle of the male spikes	1.2–2.0 cm	≤ 1.0 cm	1.0–1.5 cm	3.0–5.0 cm
Stamens	4–6	6–8	3–6	5–6
Anther	Pubescent	Glabrous	Pubescent	Pubescent
Fruit	Subglobose, 3.2–3.5 × 3–3.5 cm	Oblate, 2.0–2.5 × 2.1–2.5 cm	Obovate, 3.0–3.5 × 2.5–3.5 cm	Subglobose, 2.2–2.4 × 2.6–3.0 cm
Husk	Smooth	Smooth	Smooth	With slight wings
Shell	With 4 faint, longitudinal ridges	Smooth	With 4 faint, longitudinal ridges	With 4 faint, longitudinal ridges
Lacunae in nut shell	Absent	Present	Present	Absent
Flowering	March to April	March to April	March	April to May
Fruiting	September	August to September	August	September

### 2.2 Plastome Sequencing, Assembly, and Annotation

Total genomic DNA was extracted from silica‐dried leaf using a modified CTAB procedure (Doyle and Doyle 1987). DNA purity and concentration were determined using a NanoDrop spectrophotometer and a Qubit fluorometer, respectively. Shotgun libraries with an average insert size of approximately 350 bp were constructed and sequenced as paired‐end (150 bp) reads on the Illumina NovaSeq 6000 platform (NovoGene, Beijing, China). The low‐quality reads were filtered using Trimmomatic v.0.32 (Bolger et al. 2014), generating approximately 4 Gb clean reads. GetOrganelle v1.7.5 (Jin et al. 2020) was employed to assemble the plastome based on clean data with default settings. To verify the correctness of assembly, the assembly result was checked in Bandage v0.8.1 (Wick et al. 2015). The plastid genome was annotated using CPGAVAS2 (Shi et al. 2019) and Geseq (Tillich et al. 2017), with *C. tonkinensis* (MW368388) and *C. kweichowensis* (ON782381) as references. The assembled plastome genome sequence has been deposited in CNGB (CNPO008860).

### 2.3 Plastome Structure and Features Analyses

To characterize the structure features, the expansion and contraction of inverted repeat (IR) regions were detected by online program CPJSdraw (Li et al. 2023). Simple sequence repeats (SSRs) were identified in MISA‐web (Beier et al. 2017), with parameters set to ten, five, four, three, three, and three for mono‐, di‐, tri‐, tetra‐, penta‐, and hexa‐nucleotides, respectively. The repeat distribution in plastome was counted by CPStools v2.5 (Huang et al. 2024). Additionally, Relative Synonymous Codon Usage (RSCU) was calculated using CodonW v1.4.4 (Peden 2000) to evaluate codon usage preference. Also, the Effective Number of Codons (ENC) and GC content of the synonymous third codon positions (GC3s) were used to assess codon usage patterns.

### 2.4 Phylogenetic Analyses

A total of 35 plastid sequences were used for phylogenetic analyses, comprising 28 sequences from 22 *Carya* species, six representatives from five other genera of Juglandaceae, and *Morella rubra* (Myricaceae) as outgroup (Li et al. 2025; Xi et al. 2022). The alignment of the complete plastome sequence was carried out using MAFFT v7.490 under auto strategy (Kazutaka and Standley 2013), followed by manual adjustment. The phylogenetic tree was constructed by maximum likelihood (ML) method with IQtree v.1.6.8 (Nguyen et al. 2015), and Bayesian inference (BI) with MrBayes v.3.2.7a (Ronquist et al. 2012). The best‐fit model was selected with ModelFinder v.2.2.0 (Kalyaanamoorthy et al. 2017). ML analysis was performed 1000 standard bootstraps under GTR + G4 + F model. BI analysis was performed under the GTR + G model with 10,000,000 generations, implemented in two independent runs, each consisting of four Markov chain Monte Carlo (MCMC) chains, starting from random trees and sampling one tree every 1000 generations. Based on chloroplast genome data after alignment, genetic distance was calculated using the R package APE. The resulting phylogenetic tree was visualized and edited using iTOL (https://itol.embl.de/).

## 3 Results

### 3.1 Taxonomic Treatment

**FIGURE 2:**
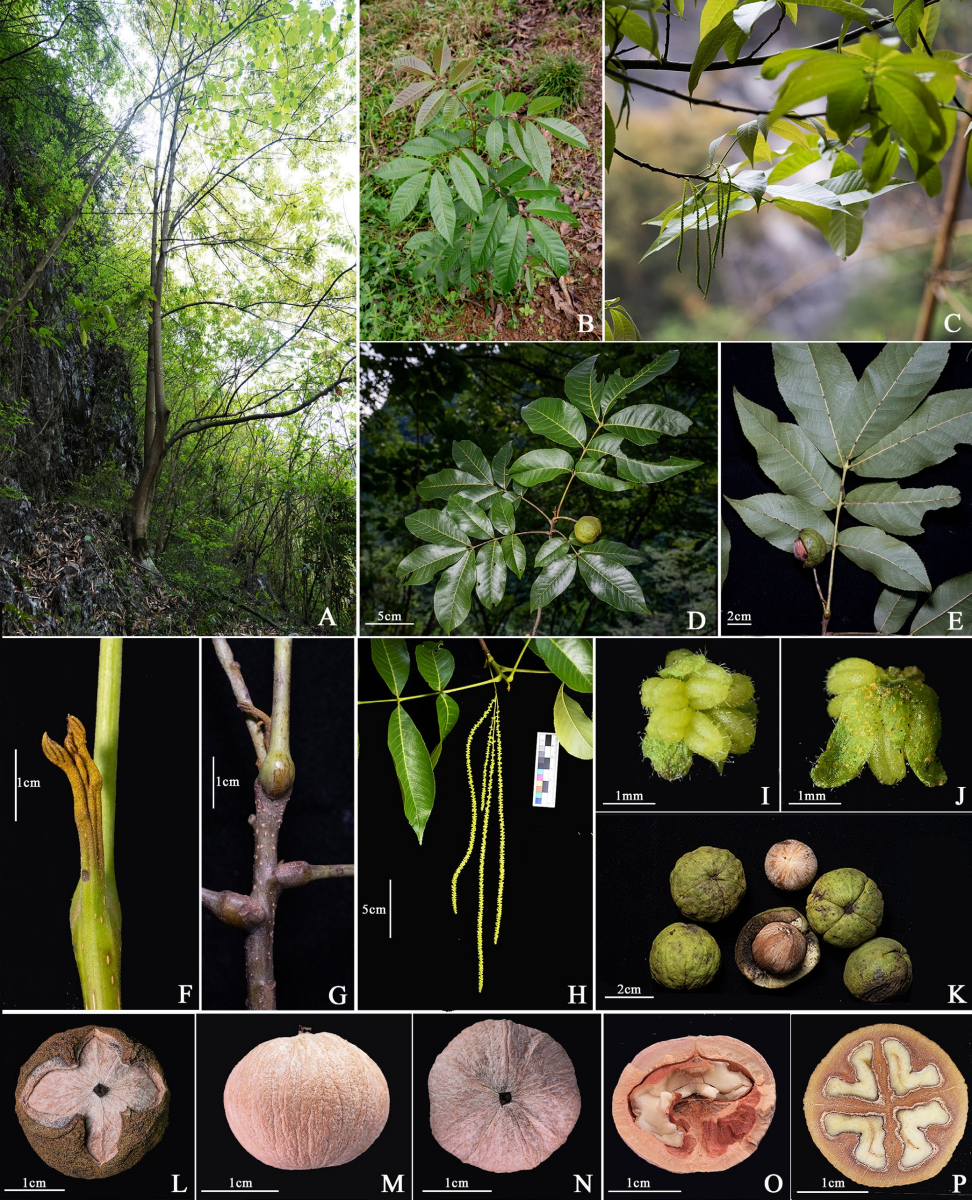
Images of *Carya luodianensis*. (A) Habitat. (B) Sapling. (C) Branch with inflorescence. (D) Upper surface of leaves and fruiting branch. (E) Lower surface of leaves. (F) terminal buds. (G) Petiole at base and branchlets. (H) Male spikes. (I) Male flower. (J) Bracts. (K) Fresh fruit. (L) Dried fruit. (M) Shell, lateral view. (N) Shell, vertical view. (O) Longitudinal section of nut. (P) Transversal section of nut.

***Carya luodianensis* Y.B. Yang & M.T. An, sp. nov. (Figures** **2** **and** **3** **)**.

**FIGURE 3:**
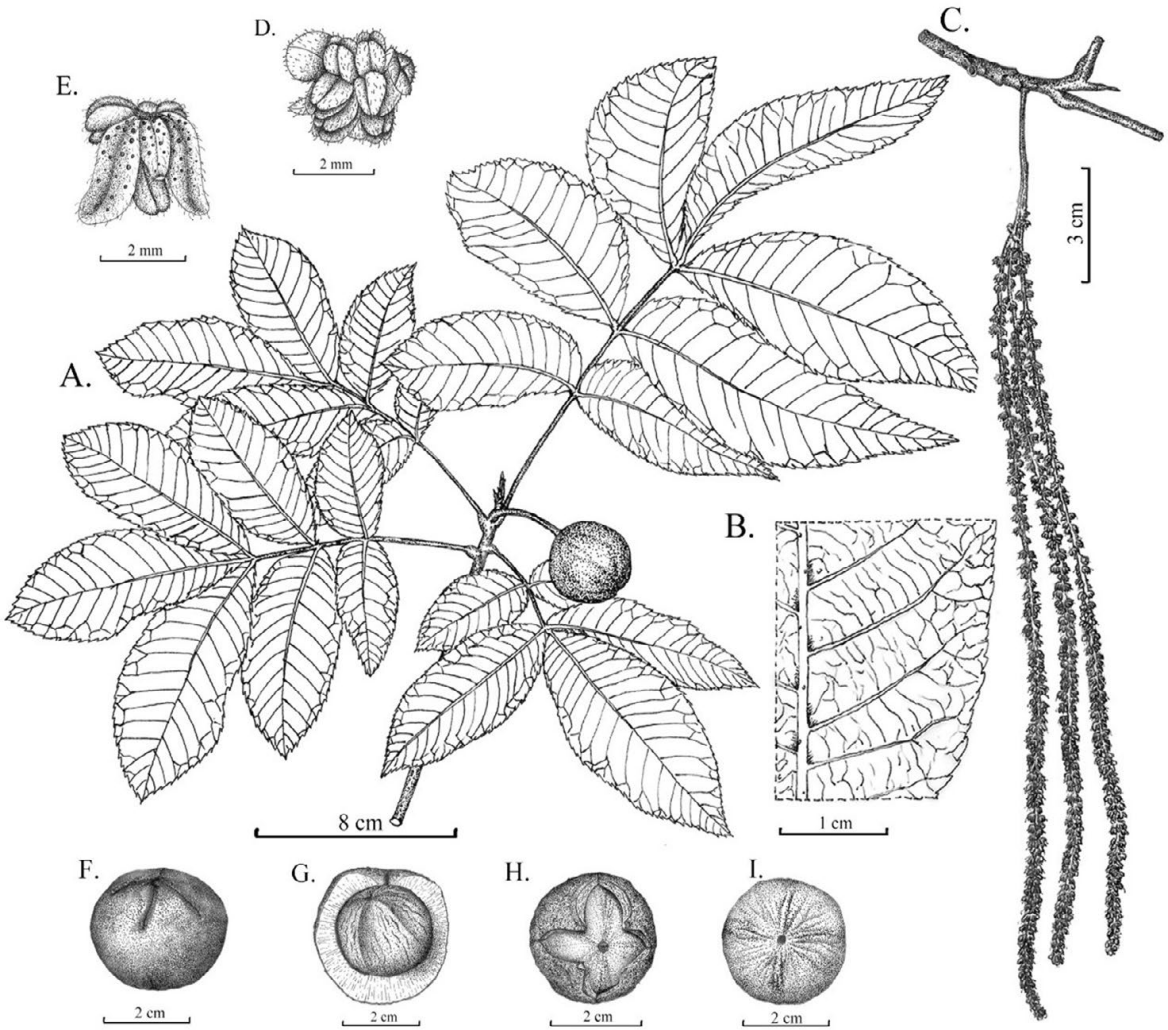
Line drawing of *Carya luodianensis*. (A) Fruiting branch. (B) Lower surface of leaves. (C) Male spikes. (D) Male flower. (E) Bracts. (F) Fruit, front view. (G) Husk and shell, front view. (H) Dried fruit, vertical view. (I) Shell, vertical view. Drawn by Bing Yang from the holotype. Phenology. Flowering from March to April, fruiting September.

**Diagnosis**. The new species is morphologically similar to *C. kweichowensis*, but can be distinguished by its rusty brown buds (vs. brownish black), 5–7 leaflets (vs. only 5 leaflets), more lateral veins (12–21 vs. 11–13 pairs), pubescent anther (vs. glabrous), nut shell with four faint longitudinal ridges (vs. smooth), and absence of lacunae in the nut shell (vs. lacunae present).

**Type**. CHINA. Guizhou Province, Luodian County, Bianyang Town, under the broad‐leaved forests in limestone montane areas, 25°33′24″ N, 106°36′34″ E, alt. 880 m, 12 September 2023, Yan‐Bing Yang, Xian‐Hua Long, Xian Jiang, LD202303 (holotype: GF, isotypes: GF, GZAC, KUN).

**Description**. Tree, semi‐evergreen. Bark gray, with shallow longitudinal ridges. Twigs green, densely covered with rusty yellow glands. Branchlets brown, with densely scattered lenticels. Terminal buds naked, rusty yellow, with densely set rusty glands. Leaf length 18–40 cm (incl. petiole), petiole and axes shortly pubescent, with scattered glands. Leaflets 5–7; lateral leaflets sessile or subsessile, terminal petiolules ca.5 mm; terminal leaflets 15–20 × 4.5–6.5 cm; lateral leaflets 8–18 × 3.5–5 cm; leaf blades thick papery, elliptic to elliptic‐lanceolate, base asymmetrically cuneate, apex acuminate, with serrate margins; adaxial light green; abaxial pale green, mid‐rib raised, pubescent in axils of secondary veins, lateral veins 12–21 pairs. Male spikes 3–4, 8.0–25 cm; peduncle flat, 1.2–2 cm, bracts 2, lanceolate, 6–9 mm, densely covered with rusty glands. Male flowers sessile, green, 1.0 × 1.5 mm; bract 1; bracteoles 2, or small clefts 3, ca.1.5 mm long, oval, hairy outside and at edge, glandular; stamens 4–6 per flower, green, oblong, with longitudinal grooves, with short hairs. Female flowers not observed. Fruits subglobose, 2.5–3 × 3–3.5 cm, peduncle long 3–4 cm, with grooves at the top; husk wingless, smooth, sparely orange‐glandular, 4–5 mm thick, cracks into 4 petals when dried; shell subglobose, with 4 faint, longitudinal ridges, apex slightly convex, 1.5–2.5 mm thick, 4 chambered at base, lacunae absent in the wall.

**Etymology**. The species epithet “luodianensis” is derived from the type locality of this species. Its Chinese name is given as 罗甸山核桃 (Pinyin: luó diàn shān hé táo).

**Distribution and Ecology**. *Carya luodianensis* is currently known only from its type locality. It grows in broad‐leaved forest at an altitude of 880 m, and typically grows with *Nephrolepis cordifolia* (L.) C. Presl, *Pteroceltis tatarinowii* Maxim., *Jasminum nervosum* Lour., *Alchornea trewioides* (Benth.) Müll. Arg., *Phanera championii* Benth., *Bambusa* sp., *Smilax* sp., and *Pistacia weinmanniifolia* J. Poiss. ex Franch.

**Conservation Status**. Due to insufficient field investigation and limited available data, an accurate assessment of the conservation status of this new species cannot currently be made. According to the criteria established by the International Union for Conservation of Nature (IUCN), this species is currently classified as Data Deficient (DD) (IUCN Standards and Petitions Committee 2024). However, seeds collected in 2023 have been successfully germinated, and over 10 saplings have been propagated and transplanted to the Arboretum of Guizhou Academy of Forestry for ex situ conservation.

### 3.2 Characteristics of Plastid Genomes

#### 3.2.1 General Features

**FIGURE 4:**
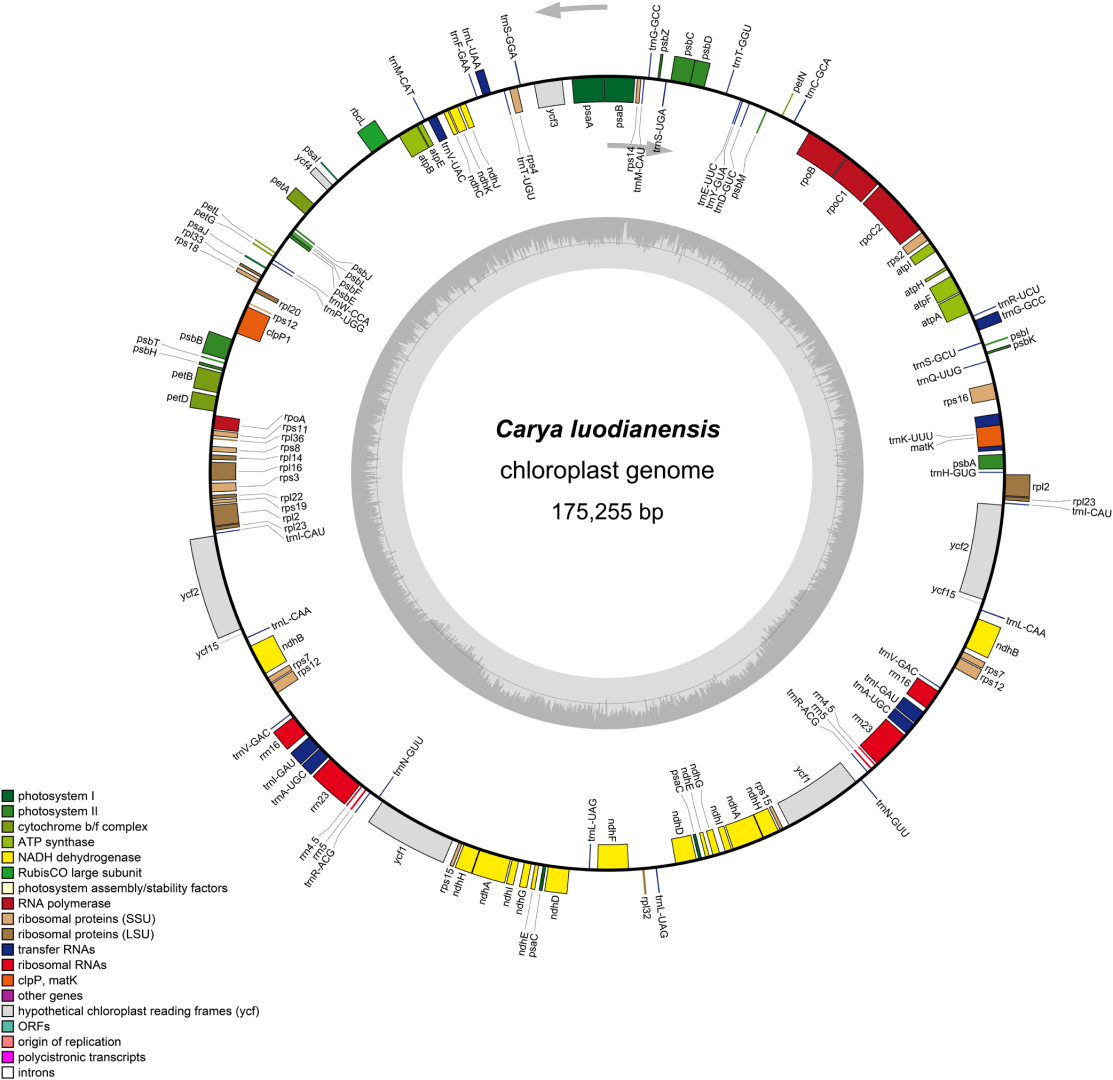
Circular maps of plastid genome of *Carya luodianensis*. Genes inside the circle are transcribed clockwise, while those outside of it are transcribed counterclockwise. The genes are color‐coded by function, with darker gray and lighter gray representing GC content and AT content, respectively.

The complete plastid genome of *C. luodianensis* exhibited a typical quadripartite structure with 175,255 bp in size, comprising a large single copy (LSC) region of 89,790 bp, a small single copy (SSC) region of 3553 bp, and two inverted repeat (IR) regions of 40,956 bp (Figure 4). GC content in the *C. luodianensis* plastome presented uneven distribution, with 38.26%, 33.81%, and 27.47% in the IR, LSC, and SSC regions, respectively. In total, 112 unique genes were annotated, consisting of 78 protein‐coding genes (PCGs), 30 tRNA genes, and four rRNA genes (Table 2). Among these, 17 PCGs, eight tRNA genes, and four rRNA genes were located within the IR regions, with two copies of each. A total of 18 genes with introns were observed, of which *clpP1*, *rps12*, and *ycf3* genes contained two introns and others possessed a single intron.

**TABLE 2:** Gene content of *Carya luodianensis* plastome.

Category	Gene group	Gene name
Photosynthesis	Subunits of photosystem I	*psaA*, *psaB*, *psaC*(2), *psaI*, *psaJ*
	Subunits of photosystem II	*psbA*, *psbB*, *psbC*, *psbD*, *psbE*, *psbF*, *psbH*, *psbI*, *psbJ*, *psbK*, *psbL*, *psbM*, *psbT*, *psbZ*
	Subunits of NADH dehydrogenase	ndhA*(2), ndhB*(2), *ndhC*, *ndhD*(2), *ndhE*(2), *nd*hF, *ndhG*(2), *ndhH*(2), *ndhI*(2), *ndhJ*, *ndhK*
	Subunits of cytochrome b/f complex	*petA*, petB*, petD*, *petG*, *petL*, *petN*
	Subunits of ATP synthase	*atpA*, *atpB*, *atpE*, atpF*, *atpH*, *atpI*
	Large subunit of rubisco	*rbcL*
Self‐replication	Proteins of large ribosomal subunit	*rpl14*, rpl16*, rpl2*(2), *rpl20*, *rpl22*, *rpl23*(2), *rpl32*, *rpl33*, *rpl36*
	Proteins of small ribosomal subunit	*rps11*, rps12**(2), *rps14*, *rps15*(2), rps16*, *rps18*, *rps19*, *rps2*, *rps3*, *rps4*, *rps7*(2), *rps8*
	Subunits of RNA polymerase	*rpoA*, *rpoB*, rpoC1*, *rpoC*2
	Ribosomal RNAs	*rrn16(2), rrn23(2), rrn4.5(2), rrn5(2)*
	Transfer RNAs	trnA‐UGC*(2), trnC‐GCA, trnD‐GUC, trnE‐UUC, trnF‐GAA, trnG‐GCC, trnG‐GCC*, trnH‐GUG, trnI‐CAU(2), trnI‐GAU*(2), trnK‐UUU*, trnL‐CAA(2), trnL‐UAA*, trnL‐UAG(2), trnM‐CAT, trnM‐CAU, trnN‐GUU(2), trnP‐UGG, trnQ‐UUG, trnR‐ACG(2), *trnR‐UCU, trnS‐GCU, trnS‐GGA, trnS‐UGA, trnT‐GGU, trnT‐UGU, trnV‐GAC*(2), trnV‐UAC*, trnW‐CCA, trnY‐GUA
Other genes	Maturase	*matK*
	Protease	clpP1**
	Envelope membrane protein	*cemA*
	Acetyl‐CoA carboxylase	*accD*
	c‐type cytochrome synthesis gene	*ccsA*(2)
Genes of unknown function	Conserved hypothetical chloroplast ORF	*ycf1*(2), *ycf15*(2), *ycf2*(2), ycf3**, ycf4

*Note:* *, Gene with one intron; **, Gene with two introns; (2), Number of copies of multi‐copy genes.

#### 3.2.2 Codon Usage Bias

**FIGURE 5:**
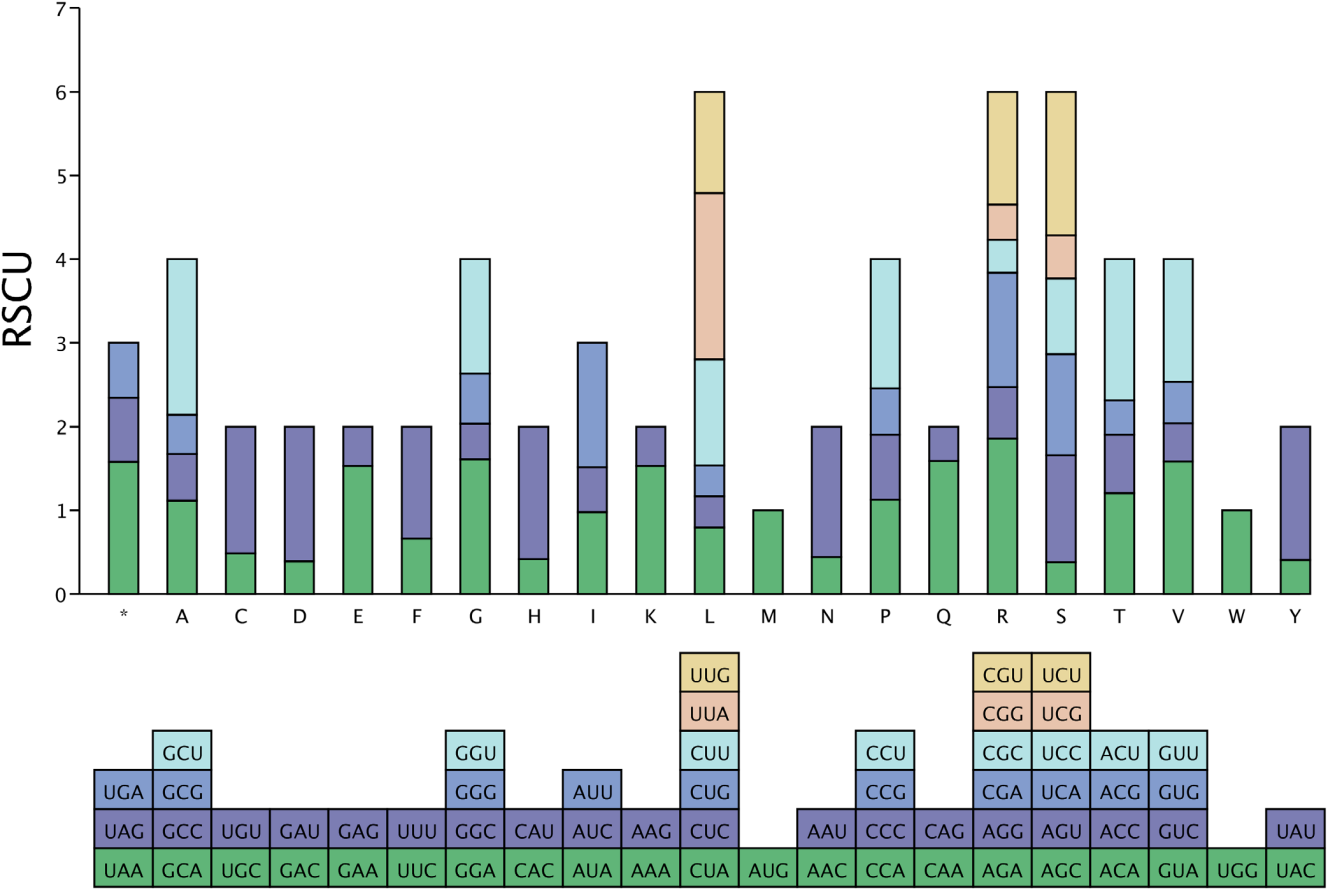
Relative synonymous codon usage of *C. luodianensis* plastome.

After removing duplicated genes, genes with non‐canonical start codons, and those shorter than 300 bp, 51 protein‐coding genes from *C. luodianensis* plastome were used to perform codon usage analyses (Table S1). The coding region was 62,187 bp in length, with an overall GC content of 37.37%. Notably, the GC content at the first codon position (45.94%) was significantly higher than that at the second (37.60%) and third positions (28.57%). The effective number of codons (ENC) ranged from 34.54 (*rps14*) to 54.23 (*ycf3*), with an average value of 47.14. A total of 20,729 codons were recognized in *C. luodianensis* plastome, encoding 20 amino acids (Table S2). Leucine‐encoding codons (2186) were the most abundant, followed by isoleucine (1862), whereas cysteine‐encoding codons were the least frequent (234). Except for methionine (Met) and tryptophan (Trp), all other amino acids were encoded by more than one codon (Figure 5). Among these, the highest and lowest relative synonymous codon usage (RSCU) values were observed for UUA (1.99) and CUG (0.36), respectively, both of which encode leucine (Leu). Moreover, the RSCU values of 30 codons were greater than 1 (1.12–1.99), with 29 of these ending in A or U. Most codons ending in G or C had an RSCU value less than 1 (0.36–0.96), with the exceptions of AUA, CUA, and UCA. For the three stop codons (UAA, UAG, and UGA), codon usage exhibited a strong preference for UAA, with an RSCU value of 1.71 (Table S2).

#### 3.2.3 Structural Variations

**FIGURE 6:**
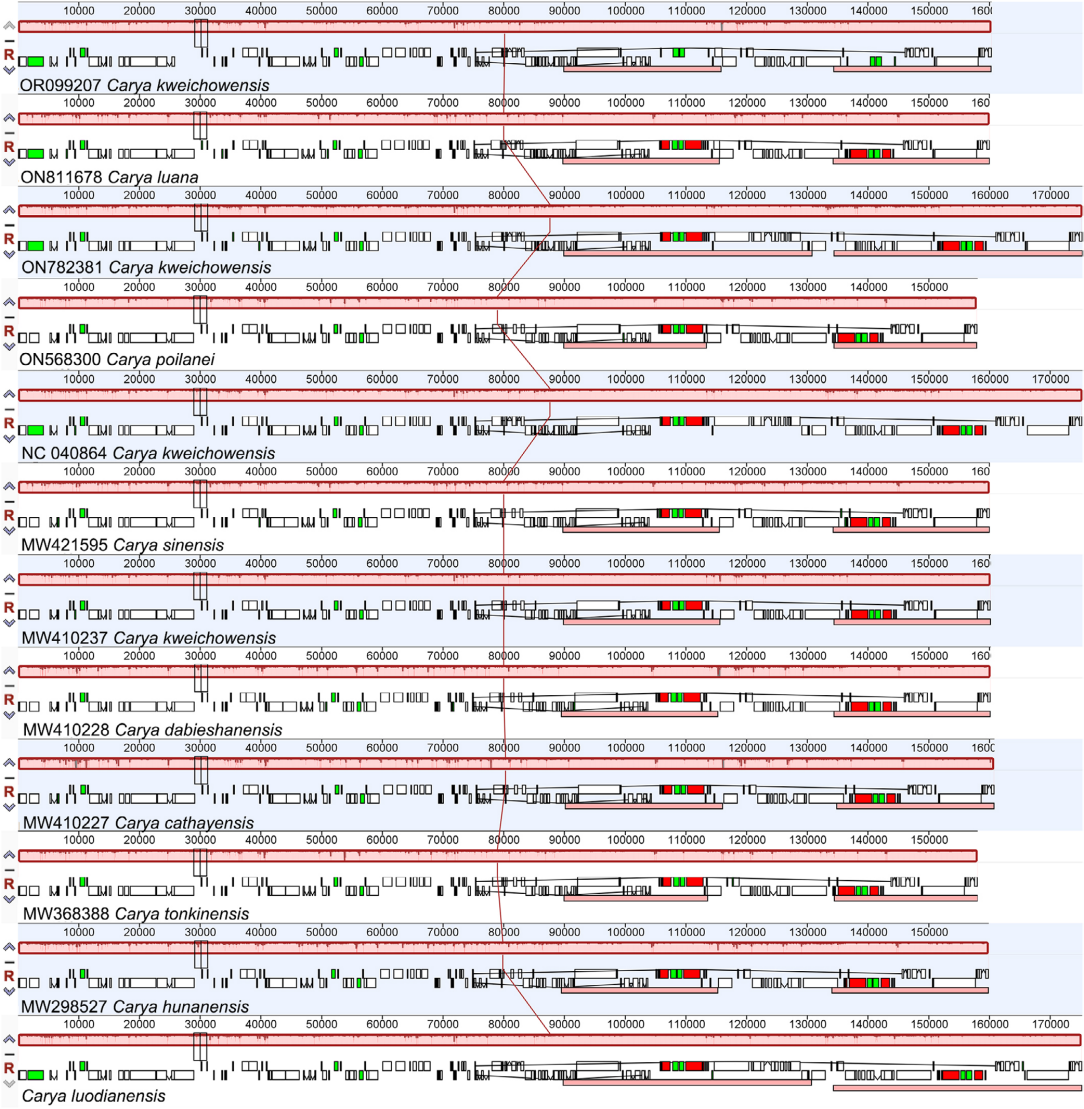
Collinearity analysis of chloroplast genomes of *C. luodianensis* and its relatives. The same color blocks represented homologous segments between different plastid genomes and were connected by lines.

**FIGURE 7:**
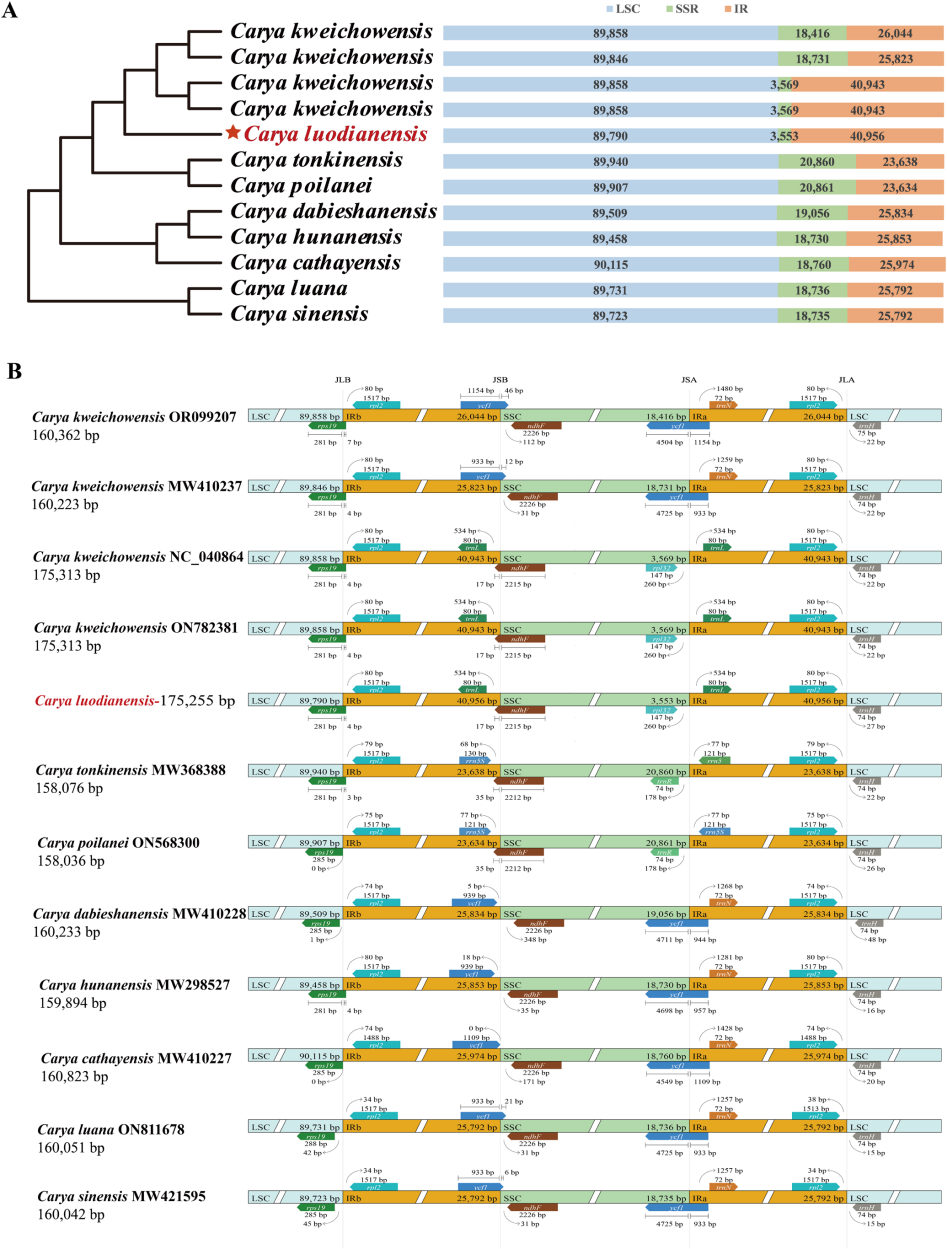
Structure variations of *C. luodianensis* plastome in size. (A) The length of different regions in *C. luodianensis* and related species. (B) The boundary analyses among LSC, IR, and SSC regions.

No gene rearrangement or inversion events were detected across *C. luodianensis* and its relatives (Figure 6), indicating that gene order was relatively conservative in plastomes. Despite this, the plastome of *C. luodianensis* demonstrated a significant expansion of the IR regions, as well as a notable contraction of the SSC region (Figure 7A). The junction of LSC and IRb was located in the intergenic region between *rps19* and *rpl2* among *C. poilanei* , *C. dabieshanensis*, and *C. cathayensis*, whereas in other species, the *rps19* gene spanned the junction (Figure 7B). The *ndhF* gene crossed the SSC‐IRb junction in two accessions of *C. kweichowensis* (NC_040864 and ON782381), *C. luodianensis*, *C. tonkinensis* , and *C. poilanei* , with 2212 bp to 2215 bp in the SSC region and 17 bp to 35 bp in the IRb region. On the other hand, the *ndhF* gene was located entirely within the SSC region in others. With the pronounced IR expansion, the SSC/IRa junction was located between *rpl32* and *trnL*, retaining an identical gene arrangement. At the LSC/IRa junction, the *rpl2* and *trnH* genes were entirely distributed within the LSC and IRa regions near the junction (Figure 7B).

#### 3.2.4 Repeat Sequence Characteristics

**FIGURE 8:**
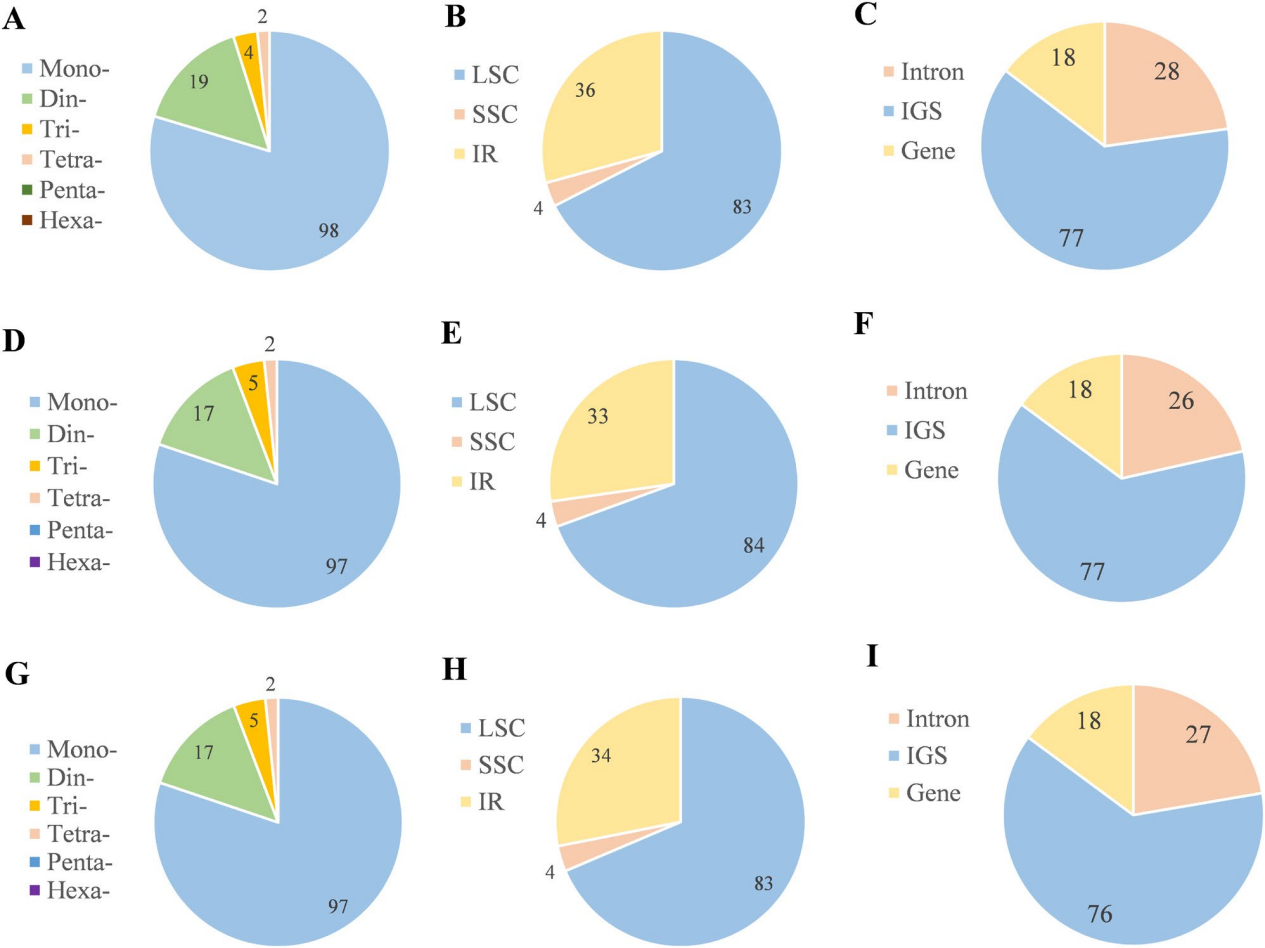
Comparisons of simple sequence repeats (SSRs) among *C. luodianensis* (A–C), *C. kweichowensis* (NC_040864) (D–F), and *C. kweichowensis* (ON782381) (G–I). (A, D, G) The number and type of SSRs. (B, E, H) The number of SSRs in LSC, SSC, and IR. (C, F, I) The number of SSRs in intron, gene, and IGS.

A total of 123 simple sequence repeats (SSRs) were detected across *C. luodianensis* plastome, including 98 mononucleotide, 19 dinucleotide, 4 trinucleotide, and 2 tetranucleotide repeats (Figure 8A). Among these mononucleotide repeats, the majority are composed primarily of A/T (98.98%). Most SSRs were distributed in the LSC region of *C. luodianensis* plastome, accounting for 67.48% (Figure 8B). These SSRs included 62.60%, 22.77%, and 14.63% in intergenic spacers (IGS), introns, and genes, respectively (Figure 8C). Furthermore, the SSRs of two plastid genomes of *C. kweichowensis* (NC_040864 and ON782381) were highly similar. A total of 121 SSRs were recognized in both plastomes (Figure 8D,G), with only slight variations in repeat distributions (Figure 8E,F,H,I).

### 3.3 Phylogenetic Position

**FIGURE 9:**
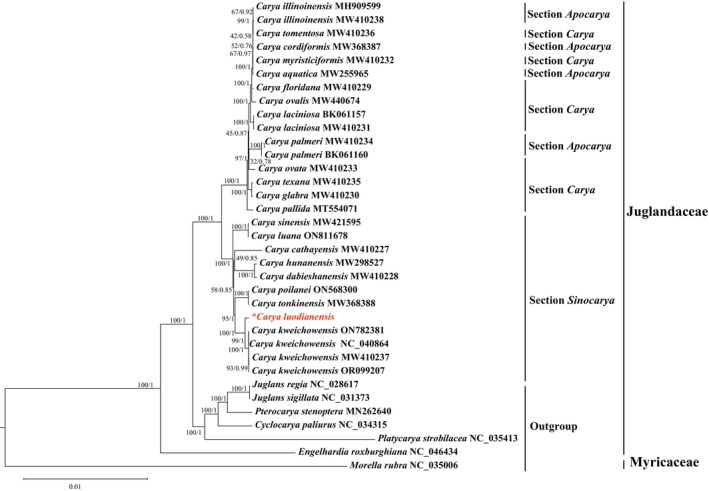
Phylogenetic tree of *C. luodianensis* and related taxa inferred from the whole plastid genomes. The bootstrap (BS) values and posterior probabilities (PP) supporting each node are displayed above the branches, and the accession numbers for GenBank are provided following the species name.

A total of 35 plastomes were applied to preform phylogenetic analyses, comprising 28 individuals representing 22 species from *Carya*, with the other seven genera from Juglandaceae and Myricaceae as the outgroups. After excluding ambiguously aligned regions, the final plastomes alignments were 158,945 bp in length, with 2348 parsimony informative sites. Phylogenetic trees were reconstructed using both the maximum likelihood (ML) method and Bayesian inference (BI) methods, which yielded identical topologies. The phylogenetic results strongly supported the monophyly of Juglandaceae (PP/BS = 1/100), the newly described species was resolved as sister to *C. kweichowensis* (PP/BS = 1/100), then together sister to *C. tonkinensis* and *C. poilanei* (PP/BS = 1/100) (Figure 9). The genetic distance between *C. luodianensis* and *C. kweichowensis* ranges from 0.00051 to 0.00052.

## 4 Discussion

To ensure accurate species delimitation, we conducted a comprehensive morphological comparison between the newly described species and morphologically similar species within the genus *Carya* (Table 1). Morphologically, *Carya luodianensis* is most similar to *C. kweichowensis*. However, it can be readily distinguished by its 5–7 leaflets (vs. 5 leaflets), a greater number of lateral veins, pubescent anthers, and nut shell with four faint longitudinal ridges. In addition, *C. luodianensis* also resembles *C. luana* and *C. tonkinensis* in possessing four faint longitudinal ridges on the shell, yet differs clearly from both. Compared to *C. luana*, *C. luodianensis* is characterized by its elliptic to elliptic‐lanceolate leaflet blades (vs. lanceolate or ovate‐lanceolate), puberulent rachis, and subglobose fruit (Xiang et al. 2023). In comparison to *C. tonkinensis* , *C*. *luodianensis* can be distinguished by its semi‐evergreen habit, larger leaves without peltate scales, fewer lateral veins, and smooth husks (Zhang et al. 2022).

Phylogenetically, *C*. *luodianensis* is sister to *C. kweichowensis*, which is congruent with morphological evidence. Furthermore, the phylogenetic results indicated that the clade comprising *C. luodianensis* and *C. kweichowensis* was sister to *C. tonkinensis* and *C. poilanei* , suggesting that the chloroplast genome is an effective marker for species identification in *Carya* (Xi et al. 2022). Taken together, both molecular and morphological evidence strongly support the taxonomic recognition of *C. luodianensis* as a distinct species.

Generally speaking, plastid genomes among land plants exhibit a high degree of conservation, with a stable quadripartite structure, conserved gene order, and similar gene content (Wicke et al. 2011). The plastid genome of *C. luodianensis* presented a typical quadripartite structure and consisted of 78 protein‐coding genes (PCGs), 30 tRNA genes, and four rRNA genes, which is similar to other *Carya* plastomes (Xi et al. 2022). In terms of codon usage pattern, the plastid genome of *C. luodianensis* was relatively conserved, with a similar A/U‐ending preference, uneven GC content, and gene‐specific ENC value. These findings indicated that gene expression is highly conserved at the plastid level, consistent with previous studies (Gu et al. 2020; Zhao et al. 2020; Peng et al. 2023; Yan, Geng, et al. 2023). However, the GC content in the inverted repeat (IR) regions of the *C. luodianensis* plastome (38.26%) was lower than that of other closely related *Carya* species (42.57%–42.89% in Xi et al. 2022), potentially due to dynamic shifts at the IR‐SC junctions. As one of the most conserved elements in plastid genomes, the expansion or contraction of the IR regions may lead to size variation, gene loss, gene duplication, and pseudogenization (Dugas et al. 2015), which serves as a crucial evolutionary indicator for resolving taxonomic relationships (Park et al. 2018; Song et al. 2022; Yan, Gu, et al. 2023). Moreover, repeated sequences, particularly simple sequence repeats (SSRs), play a crucial role in genome stability, recombination, and mutation, thereby contributing to plastid genome variability, structural rearrangements, and evolutionary dynamics (Zhu et al. 2021). Compared to most *Carya* species (Xi et al. 2022; Xiang et al. 2023), the plastid genome of *C. luodianensis* exhibited a significant expansion of the IR region (40,956 bp) and contraction of the SSC region (3553 bp). With the IR expansion, the genomic size (175,255 bp) and gene content (141) of *C. luodianensis* were more than those of other *Carya* species, such as *C. tonkinensis* (158,076 bp and 131 genes) and *C. luana* (160,051 bp and 135 genes). The transfer of several genes originally located in the SSC region to the IR region has resulted in an expansion of duplicated gene content. For instance, previous studies have reported that 18 genes were located within the IR regions across 19 *Carya* plastomes (Xi et al. 2022), whereas 29 duplicated genes were identified in the *C. luodianensis* plastome. Although the two *C. kweichowensis* plastid genomes exhibited a similar phenomenon in structural variations, *C. luodianensis* displayed slight differences in the number and distribution of SSRs compared to both. Overall, the distinct plastid structure of *C. luodianensis*, characterized by notable variations in IR expansion, SSC contraction, and SSR distribution, underscores its evolutionary divergence from other *Carya* species. These structural differences may reflect the unique genomic adaptations (Daniell et al. 2016; Do and Kim 2017; Gao et al. 2019), which suggests that further comparative plastome studies across the genus could provide valuable insights into the evolutionary divergence and adaptive strategies for *Carya* species.

## Author Contributions

**Yan‐Bing Yang:** conceptualization (equal), methodology (equal), visualization (lead), writing – original draft (lead). **Chong‐Yi Yang:** conceptualization (equal), visualization (supporting), writing – original draft (equal). **Wei‐Hao Yao:** conceptualization (supporting), writing – original draft (supporting). **Bing Yang:** visualization (supporting), writing – original draft (equal). **Lang Huang:** visualization (supporting), writing – original draft (supporting). **Ming‐Tai An:** conceptualization (lead), funding acquisition (lead), methodology (equal), writing – review and editing (lead). **Guo‐Bing Jiang:** methodology (equal), writing – original draft (supporting). **Hong Luo:** methodology (supporting), writing – review and editing (equal). **He Li:** conceptualization (lead), writing – original draft (supporting).

## Conflicts of Interest

The authors declare no conflicts of interest.

## Supporting information


**Table S1:** Effective number of codons (ENC) and GC content in *Carya luodianensis* plastome.
**Table S2:** Relative synonymous codon usage (RSCU) of *Carya luodianensis* plastome.

## Data Availability

The sequence data of *Carya luodianensis* has been deposited in CNGB under accession number CNP0008860, and the voucher specimens of the new species were deposited in GF, KUN, and GZAC.
