# Correction to “*Carya luodianensis* (Juglandaceae), a New Species From Guizhou Province, Southern China, Revealed by Morphological and Plastid Evidence”

**DOI:** 10.1002/ece3.73350

**Published:** 2026-03-31

Yang, Y.‐B., C.‐Y.Yang, W.‐H.Yao, et al. 2026. “*Carya luodianensis* (Juglandaceae), a New Species From Guizhou Province, Southern China, Revealed by Morphological and Plastid Evidence.” *Ecology and Evolution* 16, no. 3: e73047. 10.1002/ece3.73047.

In the published article, the following funding information should be added:

**Funding Information:** This work was supported by the Survey and Collection of Germplasm Resources for *Carya kweichowensis* (2024‐ZM‐22); the Forestry Science Project of Guizhou Province of China (QLKHJZ[2025]06H); the Survey and Assessment of Newly Added National Key Protected Wild Plant Resources in Guizhou Province (Third stage) (MCHC‐ZD20242057); and the Science and Technology Planning Project of Guizhou Province (QKHFQ[2023]009).

We apologize for these errors.

